# Biting behaviour of *Simulium damnosum *complex and *Onchocerca volvulus *infection along the Osun River, Southwest Nigeria

**DOI:** 10.1186/1756-3305-3-93

**Published:** 2010-10-07

**Authors:** Monsuru A Adeleke, Chiedu F Mafiana, Sammy O Sam-Wobo, Ganiyu O Olatunde, Uwem F Ekpo, Olaoluwa P Akinwale, Laurent Toe

**Affiliations:** 1 Molecular Parasitology Laboratory, Public Health Division, Nigerian Institute of Medical Reseach, P.M.B 2013, Yaba, Lagos, Nigeria; 2 Executive Secretary Office, National University Commission, Abuja, Nigeria; 3 Department of Biological Sciences, University of Agriculture, Abeokuta, Nigeria; 4 Department of Crop Protection, University of Agriculture, Abeokuta, Nigeria; 5 Molecular Biology Laboratory, WHO/Multidisease Surveillance Centre, Ouagadougou, Burkina Faso

## Abstract

**Background:**

Studies on biting behaviours and infectivity status of insect vectors are pre-requisites in understanding the epidemiology of the vector- borne diseases and planning effective control measures. A longitudinal study was carried out to investigate the transmission index of *Simulium damnosum *complex species along Osun River, South Western Nigeria. Adult flies were collected on human attractants from 07:00 to 18:00 hours for two consecutive days from February 2008 to June 2009 at three communities: Osun Eleja, Osun Ogbere and Osun Budepo. The infectivity rate was determined by dissection and Polymerase Chain Reaction amplification (PCR) of 0-150 genes of *Onchocerca *parasite using the pool screening technique.

**Results:**

The results indicated that the majority of the flies collected at the three sampling points were nulliparous as they accounted for 53.90%, 57.86% and 59.58% of the flies dissected at Osun Budepo, Osun Ogbere and Osun Eleja, respectively. The parous rate was higher during the dry season than the wet season but the difference was not statistically significant (*p *< 0.05). The biting activity of the parous flies showed two peaks at Osun Budepo and three peaks at Osun Eleja and Osun Ogbere. Of the 1,472 flies dissected and 1,235 flies screened by molecular method, none was infected with *Onchocerca *parasite at the three sampling points however the annual biting rates at the three communities were higher than 1,000 considered as tolerable value for a person living in an onchocerciasis zone by Word Health Organization.

**Conclusion:**

The study has provided the baseline data for further study on onchocerciasis transmission dynamics and the need to intercept man- simuliid vector contact at the study area.

## Background

In West Africa, members of *Simulium damnosum *complex are the only known vectors of human onchocerciasis [1]. The disease constitutes a serious public health problem of considerable magnitude in many riverine areas of West Africa. The disease is prevalent in 35 countries of the world of which 28 are in Africa and Nigeria accounts for one quarter of the global infection [2]. The current strategy of controlling the disease in Africa relies mostly on the annual chemotherapeutic treatment of the endemic communities through mass distribution of Ivermectin. Ivermectin has been proved to have microfilaricidal properties and substantially reduced the disease burden in many affected communities [3,4].

Humans acquire infections after being bitten by black flies of the *S. damnosum *species complex carrying infective larvae of *Onchocerca volvulus *[4]. Therefore, surveillance on infectivity status of the *Simulium *vectors biting along the river systems is a vital component in monitoring the transmission level of onchocerciasis which thus gives a non-invasive method of measuring the success of the control measures [5]. The classic method of capture and dissection of adult flies for infection has been the traditional technique of monitoring the dynamics of vector infectivity. Different infectivity rates can be found at different sites and it is recognized that different cytospecies of *S. damnosum *complex living in different biotypes may carry heavier *O. volvulus *infective worm loads than others [5-7].

In recent time, molecular technique has been explored as an adjunct to dissection in estimating the infectivity of adult female flies caught in the wild [4]. The O -150 PCR pool screen technique can detect a single infected fly in pools containing up to 100 flies [8]. A mathematical model to calculate prevalence of infection in the black fly population based on the size of the pools screened and on the percentage of the negative pools found [8]. Moreover, a method for the mass separation of heads and bodies of black flies was subsequently developed [4,9]. This method allows separate estimates of the prevalence of the parasites in the head capsule (representing L3 of from human or other vertebrate infection stage) and the rest of the body (representing the earlier developmental stages, i.e the L1 and L2 larvae). The field studies in Africa and Mexico showed that the 0-150 pool screen PCR assay generated the estimates of the prevalence of infected and infective flies in the vector population that were indistinguishable from those obtained by traditional dissection of the flies [4,10].

Osun river is one of the rivers in southwestern Nigeria where intense biting of black flies is being experienced by the surrounding communities but little is known on the vectorial capacity of the biting species. As part of longitudinal studies to determine the entomological profile of onchocerciasis transmission along River Osun where *S. soubrense *beffa had been identified as the predominant biting species [11], we utilized the dissection and pool screening techniques to investigate the transmission *O. volvulus *by *Simulium damnosum *s.l along River Osun, Southwestern Nigeria.

## Materials and methods

### Study area

The study was conducted along Osun river system, South Western Nigeria. River Osun lies on the latitude 08°02'N and 06°30'N; longitude 05°10'N and 03°25'E in the forest zone of Nigeria and flows through Osun, Oyo and Ogun States before emptying its water into Atlantic Ocean. Both primary forest vegetation and secondary forest vegetation (Derived savanna) are commonly seen along the river course in Osun and Oyo States but the rain forest is the largest ecological zone along Ogun State. There are over 20,000 inhabitants living along the river and the majorities are farmers by occupation. The study area has two seasons; the wet season (April to October) and the dry season (November to March). Three catching points; Osun Eleja (derived savanna), Osun Budepo (rainforest) and Osun Ogbere (rainforest) were selected along the river course. Osun Eleja is located on latitude 07'16°N; longitude 04'08°E while Osun Budepo is located on latitude 07'04°N; longitude 04'08°E. Osun Ogbere is located on the latitude 06'76°N; longitude 04'13°E. The three communities are first line communities along the river system and no form of onchocerciasis control had ever been carried out in the communities, though the residents used to receive ivermectin treatment in adjoining endemic communities. Only Osun Eleja was classified as mesoendemic by National rapid epidemiological mapping (REMO) conducted in 1995, the two other communities were considered hypoendemic but shared boundary with endemic communities in Oyo and Osun States.

### Entomological methods

The biting adult females of *S. damnosum *s.l were collected using consented fly attractants. Two adult fly catchers were positioned near the bank of the river in each of the sampling sites. The fly catching period was organized for two consecutive days at each of the sampling points fortnightly. The collectors captured flies alternately between 07:00 to 18: 00 hours every sampling week. The flies caught for the first day of every sampling week were kept for dissection while the flies caught on the second day of sampling were preserved in 80% alcohol for pool screening. The fly collectors sat at the bank of the river and exposed the lower part of their legs. Any black fly coming to perch for a blood meal on the exposed parts was caught before it fed by inverting a small plastic tube over it and the cap of the tube was then immediately replaced. The tubes were pooled according to the hour of collection in each location. The collection of the flies was carried out between February 2008 and January 2009 at Osun Budepo and Osun Ogbere and between July 2008 and June 2009 at Osun Eleja.

### Dissection of the Flies

The flies caught on the first day of every sampling week were dissected for parity and *Onchocerca *parasite. The ovaries of the dissected flies were stretched and classified as parous or nulliparous after observing other characters like absence or presence of fat bodies and the colour of malphigian tubules [12,13]. The parous flies were dissected further for infection, while the nulliparous flies were discarded. The head, thorax and abdomen of all parous flies were dissected further for different stages of *O. volvulus *larvae.

### Pool screening for fly infectivity

As an adjunct to dissection, the flies preserved in 80% ethanol were screened for *Onchocerca *parasite using pool screening technique [4,8]. The heads of the flies were separated from the body using manual cutting procedure. The heads were aliquoted into pools of twenty and the DNA was extracted and purified by strepavidin magnetic dynabeads (Invitrogen).

### PCR Amplification of O-150 repeates

5 μl of the DNA template was used in PCR amplification. The PCR amplifications were carried out in a total volume of 50 μl in a solution containing 10× of PCR buffer, 2 Mm of dNTPS, 20 μM of each of 0150-1632 5'GATTYTTCCGRCGAANARCGC3' and 0150-1633 5' GCNRTRTAAATNYGNAATTC3' primers and 1 unit of Taq polymerase (Roche Diagnostics, Indianapols, IN). The PCR cycling conditions consisted 5 cycles of 95°C for 1 minute, 37°C for 2 minutes and 72°C for 30 seconds followed by 29 cycles of 94°C for 30 seconds, 37°C for I minute and 72°C for 30 seconds. The reaction was completed by incubation at 72°C for 6 minutes. 10 μl of the PCR products were separated in 3% Ethidium bromide stained agarose gel prepared with 1× TAE buffer. The gel was run at 100 V for 50 minutes and visualized with ultraviolet transilluminator.

### Statistical Analysis

The diurnal monthly data of the parous flies were pooled for hourly variation. The analysis of data showed that the data were skewed from normal distribution. For valid application of analysis, all the data were transformed by √x+0.5. The transformed data were subjected to t- test and analysis of variance (ANOVA) to determine the significant differences in biting activities of the flies at the three locations. Pool screening result was analyzed by pool screening software as previously described [8]. The monthly and annual biting rates were estimated as earlier described by [5]. The monthly biting rate is as estimation of the number of flies that will bite a person positioned at the study site in a month while annual biting rate is the number of flies that will bite a person positioned at the study site in a year.

## Results

### Biting activity of the parous flies

**Figure 1:**
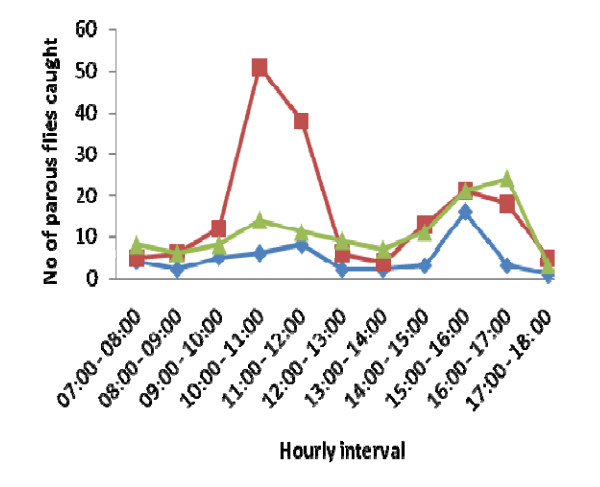
**Daily biting activity of the parous flies at the study sites**. The red line in the graph represents Osun Budepo, the blue line represents Osun Ogbere while green line represents Osun Eleja.

The diurnal biting activity of the parous flies varied significantly in each of the study sites (*p *< 0.05). At Osun Budepo, the biting density of the parous flies showed bimodal peaks (Figure 1). The first peak was observed between 10:00 - 11:00 h and the second peak occurred between 15:00 - 16:00 h. The morning peak was significantly higher than the evening peak (*p *< 0.05). At Osun Ogbere, three peaks were observed in the biting activities of the flies, 07:00 - 08:00 h, 11:00 - 12: 00 h and 15:00 - 16:00 h. The biting peak between 15:00-16:00 h was significantly higher than the remaining two peaks (*p *< 0.05). Similarly, three biting peaks were observed in the biting activities of the parous flies at Osun Eleja with 16:00 - 17:00 h peak significantly higher than 07:00 - 8:00 h and 10:00 - 11:00 h peaks (Figure 1).

### Transmission Indices of *Simulium damnosum *complex

The entomologic indices of onchocerciasis transmission at the three sites are shown in Table 1. Of the 692 flies dissected at Osun Budepo, 319 (46.10%) were parous females while 373 (53.9%) were nulliparous. None of the 319 parous flies were infected with any stage of *O.volvulus *larvae during the period of the study. The flies caught during the dry season (December to March) had higher parous rate as compared with the wet season (April to October). The month of March recorded the highest percent of parous flies (87.5%), while the least abundance was recorded in June (37.7%). The seasonal variation in monthly parity was statistically significant (*p *< 0.05). The highest monthly biting rate (MBR) was recorded in September with 3,075 bites per person per month, while the lowest MBR was recorded in January with 47 bites per person per month. The annual biting rate (ABR) was 10,493 bites per person per year. The MTP and ATP could not be evaluated since none of the flies dissected was infected. At Osun Ogbere, 67 (46.10%) out of 159 flies dissected were parous, while 92 (57.87%) were nulliparous. The percent parous rate was also significantly higher during the dry season (February to March) and the early wet season (April). February recorded the highest percent of parous flies (100%), while the lowest percent was recorded in September (30.0%). The highest monthly biting rate was recorded in September and October with 450 bites per person per month while the least monthly biting rate was recorded in December and January as no fly was caught in the two months. The annual biting rate was estimated at 2,412 bites per person per year. The MTP and ATP were 0 since none of the dissected flies was infected with *O.volvulus*.

**Table 1:** Transmission indices of onchocerciasis by *Simulium damnosum *complex along Osun River, Southwestern Nigeria

Parameters	Osun Budepo	Osun Eleja	Osun Ogbere
Total flies dissected	692	159	621
No(%) of parous flies	319 (46.10)	67 (42.13)	251 (40.4)
No (%) of nulliparous flies	373 (53.90)	92 (57.86)	370 (59.58)
Month(%) with highest parous flies	Mar (87.5)	Feb (100)	Dec (66.7)
Month(%) with lowest parous flies	Sep (33.17)	Sep (30.0)	Jun (32.16)
No of flies infected with *O. volvulus*	0	0	0
Annual biting rate	10,494	2,412	9,419
Maximum monthly biting rate	3,075 (Sep)	450 (Sep&Oct)	2,250 (Jun)
Minimum monthly biting rate	47 (Jan)	0 (Dec&Jan)	28 (Feb)

Of 621 flies dissected at Osun Eleja, 251 (40.4%) flies were parous. Majority of flies collected during the dry season (January to March) were also parous as compared with the wet season (April to October). The highest percent of parous flies was recorded in December (66.7%), while the lowest was recorded in June (32.16%). There was a significant difference in the parity of the flies over the study period (*p *< 0.05). The highest monthly biting rate was recorded in June with 2,250 bites per person per month, while the lowest monthly biting rate was recorded in February with 28 bites per person per month. The annual biting rate was 9,419 bites per person per year. None of the flies dissected was infected with any stage of *O. volvulus *larvae and the MTP and ATP were estimated as zero.

The result of the fly infectivity by pool screening is presented in table 2. None of the 682, 490 and 63 flies screened for infectivity using molecular tool was positive for *Onchocerca *parasite at Osun Budepo, Osun Eleja and Osun Ogbere, respectively. When the data were subjected to statistical analysis by pool screening software, zero infectivity rate was obtained for the three sites. However, the pool screening software showed a risk rate of onchocerciasis transmission of 2.73 × 10^-3^, 3.68 × 10^-3 ^and 2.37 × 10^-4 ^for Osun Budepo, Osun Eleja and Osun Ogbere, respectively.

**Table 2:** The *Onchocerca volvulus *infectivity status of *Simulium damnosum *complex by pool screening method along Osun River, Southwestern Nigeria

Catching points	No of flies Examined	Pool size (no of pools)	Infectivity rate (95% C.I)
Budepo	682	20 (35)	0 (0-0.00273)
Eleja	490	20 (26)	0 (0-0.00368)
Ogbere	63	20 (4)	0 (0-0.000237)

C.I = Confidence interval

## Discussion

The results from this study showed that there is significant variation in daily biting activity of *S. damnosum *s.l at the study area. Apart from Osun Budepo, the three biting peaks observed at Osun Ogbere and Osun Eleja dissented from bimodal peaks reported in other endemic communities in Nigeria [5,14-18]. The three biting peaks could plausibly indicate the variation in biting period of different species of *S. damnosum s.l *at the study area or possibly be as result of differential variation in environmental conditions at different points within the same locality. However, the later factor would have accounted for the variation since the same species of *S. damnosum *s.l exist at the three communities [11]. During the course of the study, there were instances in which rain disturbed catching activity at a site while it was not raining in other sites and vice-versa. Similar observation had recently been reported in Southwestern Nigeria [19]. The biting peaks of the parous flies were observed between 10:00 - 11:00 h and 15:00 - 16:00 h at Osun Budepo and between 07:00 - 08:00 h, 11:00 - 12:00 h and 15:00 - 16:00 h at Osun Ogbere and Osun Eleja. This is in agreement with the previous results that host seeking activities of the parous flies usually reach peak between 10:00 - 16:00 h [15,18]. This apparent biting pattern has epidemiological implications because the biting peaks correspond almost exactly to the working habit of the people in the study area, most importantly the farmers. This correspondence between the biting activity pattern of the parous flies and the working habit of the farmers maintains an uninterrupted link in man-fly contact and the risk of disease transmission by infected parous flies. Thus, changes in the practices and attitudes of the people will markedly reduce the risk of disease transmission [14-16].

The results of the dissection showed that the majority of the flies caught at the three catching points were nulliparous. This observation is consistent with the report of [16] but contrary to the report of [15,17,18]. The high proportion of the nulliparous flies at the study communities may be a reflection of local production of the flies [18]. However, the high parous rate of the flies during the dry season which was characterized with the period of little or no rainfall and the dryness of the breeding sites at the sampling points plausibly indicate the presence of migratory flies. This is possible because black flies are reportedly known as long distance migrants [20].

The absence of infection in the dissected flies during this study may indicate low level of onchocerciasis transmission along Osun river system. Among the factors that could have accounted for the low transmission are level of microfilariae in human hosts, number of surviving microfilariae in the insects, vector-species complex and the survival rate of the flies [4,8-10,21]. It has been observed that few out of hundreds of microfilariae ingested by the flies usually survive the effects of peritrophic membrane that is secreted by the female flies after blood meals [22]. Though, the individual and community microfilarial worms were not determined in the present study, the zero infectivity level of the flies may be a reflection of low level of microfilarial load in human reservoirs. This is expected in the areas where the distribution of Ivermectin is being carried out [23]. Recent report from endemic communities in Mali and Senegal has indicated the possibility of elimination of onchocerciasis after several treatments with Ivermectin [24]. None of the study communities had been treated with Ivermectin but the residents confessed that they usually received the drug from neighbouring communities. Though, the forest group flies were the predominant flies at the study communities where the forest strain of *O. volvulus *is presumed to exist [11], further studies focusing on the aspect of vector competency and parasite-vector complexes will of great importance in understanding the epidemiology of onchocerciasis at the study area.

Moreover, the relatively high number of nulliparous flies observed in the overall flies dissected in the present study could also be a contributory factor since only aged flies have chance of transmitting the parasite. It is also pertinent to stress that the zero infectivity rate observed in all the catching points by pool screening method also corroborates the result of dissection. PCR technique is more sensitive and can detect the infection once the parasite DNA is found during amplification [9]. The high risk calculated by the software is a factor of many parameters; among which are parous rate and number and size of the pools. The higher the number and size of the pools, the lower the risk factor and the more valid is the risk value generated per thousand flies [8,9]. Since the number of the flies pool screened in each of the site was less than 6,000 recommended for endemic communities, this could have resulted in the high risk value generated by pool screening. This is a major limitation and further studies considering minimum of 6,000 flies will be of help in drawing valid conclusion on the status of onchocerciasis transmission along Osun River course.

Though, none of the flies were infected, the value of ABR recorded in each of the three sampling points during this study was far above the threshold biting rate usually considered for onchocerciasis transmission by World Health Organization (WHO). The high biting rates of the flies, especially during the beginning and late wet season are of high risk to the residents. The migration of infected human hosts with high microfilariae load could as a matter of years change the transmission pattern of onchocerciasis in the area since the potential vectors are available in high abundance. As at now, the high biting rate of the flies is of biting nuisance more than disease transmission in the study locations. The biting nuisance of the flies is also of socio-economic importance. Their painful bites could serve as mechanical entry for viruses, bacteria, protozoan and nematodes which the flies may carry on their bodies. The crawling effect of their movement in human hosts could also constitute intolerable nuisance and distraction from the work therefore reducing productivity [15].

In conclusion, the present study has generated baseline information on biting behaviours and onchocerciasis transmission indices of *S. damnosum *complex along Osun river system. Further surveillance is therefore needed at the study sites and other river systems for the establishment of current status of onchocerciasis transmission in the country. The residents of the study area also need to embrace the use of insect repellents and other personal methods to reduce man- fly contact.

## Competing interests

The authors declare that they have no competing interests.

## Authors' contributions

AMA initiated and drafted the proposal. MCF, SOS, AOP, UFE, GOO and TL made inputs to the proposal. AMA carried out both field and laboratory studies. MCF, SOS, AOP, UFE GOO supervised the field work while TL, supervised the laboratory work. TL interpreted the results. AMA drafted the manuscript and TL, MCF, SOS, AOP, GOO, UFE reviewed the manuscript.

## Ethical Approval

The approval was obtained from Ministry of Health in Oyo and Ogun States and the community heads of the study sites. Informed consent was obtained from the human attractants after the study protocol was clearly explained to them. The human attractants were treated before and after the study with Ivermectin.
